# Post-Hoc Study: Intravenous Hydration Treatment in Chinese Patients with High Risk of Contrast-Induced Nephropathy Following Percutaneous Coronary Intervention

**DOI:** 10.1038/srep45023

**Published:** 2017-03-24

**Authors:** Weijie Bei, Hualong Li, Kaiyang Lin, Kun Wang, Shiqun Chen, Xiaosheng Guo, Yong Liu, Ning Tan, Jiyan Chen, TRUST investigators, Xiangtai Yang, Xi Su, Zhimin Du, Qiutang Zeng, Zhenfei Fang, Yan Wang, Hong Jiang, Longgen Xiong, Yuqing Hou, Yong Yuan, Tianfa Li, Lang Hong, Yanqing Wu, Yin Liu, Wenhua Lin, Tiemin Jiang, Junhua Fu, Yi An, Bo Yu, Ye Tian, Yang Zheng, Bin Liu, Ping Yang, Xianyan Jiang, Hao Wang, Peng Qu, Lianqun Cui, Xueqi Li, Xiaoyong Qi, Zengcai Ma, Jifu Li, Lili Zhang, Shengquan Liu, Wenyue Pang, Yibo Li, Manguang Yang, Zheng Ji, Pitian Zhao, Lu Li, Junbo Ge, Huigen Jin, Weimin Pan, Yaoming Song, Jianmei Li, Jianming Xiao, Hanxiong Liu, Jianhong Tao, Zhongdong Wu, Buxiong Tuo, Wei Li, Yixian Xu, Zhaoqi Zhang, Yundai Chen, Lefeng Wang, Jinying Zhang, Fengling Wang, Yongping Jia, Bin Wang, Fakuan Tang, Qiang Tang, Wei Wang, Yuemin Sun, Weiqing Su

**Affiliations:** 1 Department of Cardiology, Guangdong Cardiovascular Institute, Guangdong Key Laboratory of Coronary Disease, Guangdong General Hospital, Guangdong Academy of Medical Sciences, Guangzhou, China; 2 Department of Geriatric Medicine, Fujian Provincial Hospital, Fujian Provincial Institute of Clinical Geriatrics, Fujian Provincial Key Laboratory of Geriatric Disease, Fujian Medical University, Fuzhou, China; 3 Department of Cardiology, Guangdong General Hospital, Guangzhou, Guangdong, P.R. China; 4 Department of Cardiology, Wuhan Asia Heart Hospital, Wuhan, Hubei, P.R. China; 5 Department of Cardiology, First Affiliated Hospital of Sun Yat-sen University, Guangzhou, Guangdong, P.R. China; 6 Department of Cardiology, Union Hospital Tongji Medical College Huazhong University of Science and Technology, Wuhan, Hubei, P.R. China; 7 Department of Cardiology, The Second Xiangya Hospital of the Central South University, Changsha, Hunan, P.R. China; 8 Department of Cardiology, Zhongshan Hospital of Xiamen University, Xiamen, Fujian, P.R. China; 9 Department of Cardiology, The People’s Hospital of Wuhan University, Wuhan, Hubei, P.R. China; 10 Department of Cardiology, The second affiliated hospital of Guangzhou medical University, Guangzhou, Guangdong, P.R. China; 11 Department of Cardiology, Nan Fang Hospital, Guangzhou, Guangdong, P.R. China; 12 Department of Cardiology, The People’s Hospital of Zhongshan City, Zhongshan, Guangdong, P.R. China; 13 Department of Cardiology, The affiliated Hospital of Hainan Medical College, Haikou, Hainan, P.R. China; 14 Department of Cardiology, Jiangxi Provincial People’s Hospital, Nanchang, Jiangxi, P.R. China; 15 Department of Cardiology, The Second Affiliated Hospital of Nanchang University, Nanchang, Jiangxi, P.R. China; 16 Department of Cardiology, Tianjin Chest Hospital, Tianjin, P.R. China; 17 Department of Cardiology, TEDA International Cardiovascular Hospital, Tianjin, P.R. China; 18 Department of Cardiology, The Affiliated Hospital of Armed Police Medical College, Tianjin, P.R. China; 19 Department of Cardiology, The Affiliated Hospital of Medical College Qingdao University, Qiandao, Shangdong, P.R. China; 20 Department of Cardiology, The Affiliated Hospital of Medical College Qingdao University (East Area), Qiandao, Shangdong, P.R. China; 21 Department of Cardiology, The Second Affiliated Hospital of Harbin Medical University, Harbin, Heilongjiang, P.R. China; 22 Department of Cardiology, The First Affiliated Hospital of Harbin Medical University, Harbin, Heilongjiang, P.R. China; 23 Department of Cardiology, The First Hospital of Jilin University, Changchun, Jilin, P.R. China; 24 Department of Cardiology, The Second Hospital of Jilin University, Changchun, Jilin, P.R. China; 25 Department of Cardiology, The Third Hospital of Jilin University, Jilin, Changchun, P.R. China; 26 Department of Cardiology, Qingdao Fuwai Hospital, Qindao, Shangdong, P.R. China; 27 Department of Cardiology, Dalian Central Hospital, Dalian, Liaoning, P.R. China; 28 Department of Cardiology, The Second Affiliated Hospital of Dalian Medical University, Dalian, Liaoning, P.R. China; 29 Department of Cardiology, Shandong Provincial Hospital, Jinan, Shangdong, P.R. China; 30 Department of Cardiology, The Fourth Affiliated Hospital of Harbin Medical University, Harbin, Heilongjiang, P.R. China; 31 Department of Cardiology, Hebei General Hospital, Shijiazhuang, Hebei, P.R. China; 32 Department of Cardiology, Cangzhou Central Hospital, Cangzhou, Hebei, P.R. China; 33 Department of Cardiology,Qilu Hospital of Shandong University, Jinan, Shangdong, P.R. China; 34 Fushun Central Hospital, Department of Cardiology, Fushun Central Hospital, Fushun, Liaoning, PR China; 35 Daqing Oilfield General Hospital, Department of Cardiology, Daqing Oilfield General Hospital, Daqing, Heilongjiang, PR China; 36 Department of Cardiology, ShengJing Hospital of China Medical University, Shenyang, Liaoning, P.R. China; 37 Department of Cardiology, Liaocheng Hospital, Liaocheng, Shangdong, P.R. China; 38 Department of Cardiology, No. 463 Hospital of PLA, Shenyang, Liaoning, P.R. China; 39 Department of Cardiology, Tangshan Gongren Hospital, Tangshang, Hebei, P.R. China; 40 Department of Cardiology, Yidu Central Hospital of Weifang City, Weifang, Shangdong, P.R. China; 41 Department of Cardiology, Shenzhou Hospital Affiliated to Shenyang Medical College, Shenyang, Liaoning, P.R. China; 42 Department of Cardiology, Zhongshan Hospital of Fudan University, Shanghai, P.R. China; 43 Department of Cardiology, Central Hospital of Shanghai Putuo District, Shanghai, P.R. China; 44 Department of Cardiology, Yancheng City No. 1 People’s Hospital, Yancheng, Jiangsu, P.R. China; 45 Department of Cardiology, Xinqiao Hospital, Third Military Medical University, Chongqing, P.R. China; 46 Department of Cardiology,The Second People’s Hospital of Yunnan Province, Kunming, Yunnan, P.R. China; 47 Department of Cardiology, The First Affiliated Hospital of Kunming Medical University, Kunming, Yunnan, P.R. China; 48 Department of Cardiology, The Third People’s Hospital of Chengdu, Chengdu, Sichuan, P.R. China; 49 Department of Cardiology, Sichuan Academy of Medical Sciences & Sichuan Provincial People’s Hospital, Chengdu, Sichuan, P.R. China; 50 Department of Cardiology, The Fourth Affiliated Hospital of Xinjiang Medical University, Xinjiang, P.R. China; 51 Department of Cardiology, Xi’an Air Force Hospital, Xi’an, Shanxi, P.R. China; 52 Department of Cardiology, Xi’an No. 1 People’s Hospital, Xi’an, Shanxi, P.R. China; 53 Department of Cardiology, Gansu Province Hospital of Traditional Chinese Medicine, Lanzhou, Gansu, P.R. China; 54 Department of Cardiology, Beijing Anzhen Hospital of Capital Medical University, Beijing, P.R. China; 55 Department of Cardiology, 301 Hospital (General Hospital of Chinese PLA), Beijing, P.R. China; 56 Department of Cardiology, Beijing Chaoyang Hospital of Capital Medical University, Beijing, P.R. China; 57 Department of Cardiology, The First Affiliated Hospital of Zhengzhou University, Zhengzhou, Henan, P.R. China; 58 Department of Cardiology, Henan Provincial Chest Hospital, Zhengzhou, Henan, P.R. China; 59 Department of Cardiology, The First Affiliated Hospital of Shanxi Medical University, Taiyuan, Shanxi, P.R. China; 60 Department of Cardiology, The 721th Hospital of Chinese PLA, Beijing, P.R. China; 61 Department of Cardiology, The 309th Hospital of Chinese PLA, Beijing, P.R. China; 62 Department of Cardiology, Peking University Shougang Hospital, Beijing, P.R. China; 63 Department of Cardiology, The First Affiliated Hospital of Guangzhou Medical College, Guangzhou, Guangdong, P.R. China; 64 Department of Cardiology, General Hospital of Tianjin Medical University, Tianjin, P.R. China; 65 Department of Cardiology, Lianjiang People’s Hospital, Fuzhou, Fujian, P.R. China

## Abstract

Contrast-induced nephropathy (CIN) develops after the injection of iodinated contrast media. This is a post hoc analysis of the data obtained from the TRUST study, which was a prospective, multicentre, observational study conducted to evaluate the safety and tolerability of the contrast medium iopromide in patients undergoing cardiac catheterization from August 2010 to September 2011 in China, conducted to explore the current status, trends and risk predictors of hydration treatment. The status of hydration to prevent CIN in each patient was recorded. Of the total 17,139 patients from the TRUST study (mean age, 60.33 ± 10.38 years), the overall hydration usage was 46.1% in patients undergoing percutaneous coronary intervention (PCI) and 77.4%, 51.7%, and 48.5% in patients with pre-existing renal disease, diabetes mellitus, and hypertension, respectively. The proportion of hydration use increased from 36.5% to 55.5% from August 2010 to September 2011, which was independently associated with risk predictors like older age, pre-existing renal disease, hypertension, diabetes mellitus, prior myocardial infarction, ST segment elevation MI, high contrast dose, multi-vessel disease and reduced LVEF (<45%). Overall, the usage of intravenous hydration treatment for patients with a high risk of CIN following PCI was high in China.

It is estimated that an average of 23–46% patients with coronary artery disease (CAD) develop chronic kidney disease (CKD) or end-stage renal disease as a major complication, which accounts to increase hospitalization and mortality worldwide^1^,^2^,^3^. Contrast-induced nephropathy (CIN) or contrast-induced acute kidney injury (CI-AKI) can develop after the injection of iodinated contrast media^4^. The incidence of CIN is about 3.3% in general population^5^, whereas it can be 20% and up to 50% in patients with concomitant or previous history of severe cardiac^4^ and kidney^6^ diseases, respectively. In the past decade, the number of cardiac catheterizations, including coronary angiography (CAG) and percutaneous coronary intervention (PCI), has increased by 17 and 21 fold, respectively, in China, according to the China PEACE study^7^. As per the Chinese National data of coronary intervention, there were 567,583 cases of PCI in 2015, with an annual growth rate of 10–15%^8^. This increase in PCI cases led to a rise in CIN incidences, making it the third largest cause of hospitalization for AKI, which was associated with increased cases of renal replacement treatments, cardiovascular adverse events and high healthcare costs^9^,^10^,^11^.

Strategies to prevent CIN such as identifying high-risk patients who may develop CIN and restricting them to the procedure, reducing contrast agent volume and intensifying pre-procedural intravenous saline hydration, respectively, were established. The current PCI guidelines recommend pre- and post-procedural intravenous infusion of isotonic saline as a prevention strategy^12^,^13^. Moreover, the ACC/ESC PCI practice guidelines and most clinical studies have recommended saline hydration in patients with pre-existing renal disease or kidney dysfunction undergoing CAG/PCI at a rate of 1 mL/kg/h 12 h before and 12–24 h after the procedure^12^,^13^,^14^,^15^.

According to a cross-sectional survey conducted in China, about 120 million adults have CKD (estimated glomerular filtration rate [eGFR]: <60 mL/min/1.73 m^2^)^16^ and approximately 60% of the patients undergoing PCI have CKD (eGFR <90 mL/min/1.73 m^2^)^17^. Therefore, hydration treatment is essential, especially in patients with risk factors such as kidney disease, reduced ventricular function and diabetes. However, data on the status of hydration treatment use in China are scarce. Therefore, we conducted a post hoc data analysis from the safety and toleRability of UltraviSt in patients undergoing cardiac caTheterization (TRUST) study^18^ to provide evidence on hydration use and also the risk predictors for hydration administration.

## Results

### Baseline and procedural characteristics

The baseline characteristics of the enrolled patients are given in Table 1. Of the 17,513 patients invited to participate in the survey, 374 were excluded due to missing hydration records and 17,139 patients were enrolled in the survey (mean age, 60.33 ± 10.38 years; men, 64.3%). When stratified based on the hydration status, 7901/17,139 (46.1%) patients were found to have received hydration treatment peri-procedurally. The average hydration volume in the hydration group was 1105.25 ± 631.73 mL and there was no saline hydration use in the no hydration group. There were more comorbidities in the hydration group patients than in the non-hydration group patients, such as hypertension (58.5% vs. 53.1%, *P* < 0.0001), diabetes mellitus (22.5% vs. 18.0%, *P* < 0.0001), prior MI (8.2% vs. 5.9%, *P* < 0.0001) and pre-existing renal disease based on personal health history (2.3% vs. 0.6%, *P* < 0.0001). There was also a higher patient proportion diagnosed with STEMI, unstable angina, multi-vessel disease, left main lesion and left anterior descending branch disease in the hydration group (*P* < 0.0001). Furthermore, the hydration group patients had received larger doses of the contrast agent (140.38 ± 80.59 vs. 111.49 ± 62.57 mg of iodine/mL, *P* < 0.0001). There were no significant differences between the groups in terms of age, gender and history of prior catheterization.

### Proportion of saline hydration rates in high-risk patients

**Figure 1:**
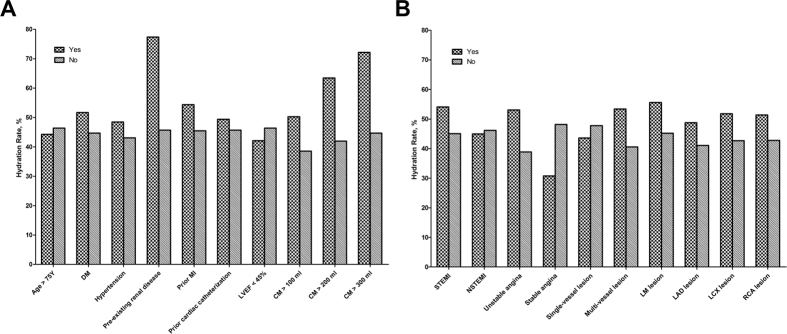
Hydration rate status of patients. (**A**) Hydration rate of patients with or without risk factors; (**B**) Hydration rate of patients with different coronary diseases. Abbreviations: DM, diabetes mellitus; MI, myocardial infarction; LVEF, left ventricular ejection fraction; CM, contrast medium; STEMI, ST segment elevation myocardial infarction; NSTEMI, non-ST segment elevation acute myocardial infarction; LM lesion, left main lesion; LAD lesion, left anterior descending branch lesion; LCX lesion, left circumflex branch lesion; RCA lesion, right coronary lesion.

The hydration rate in patients with and without risk factors is shown in Fig. 1. Hydration was significantly higher in patients with pre-existing renal disease (77.4% vs. 45.7%), STEMI (54.1% vs. 45.1%), unstable angina (53.1% vs. 38.9%) and multi-vessel disease (53.4% vs. 40.6%) (all *P* < 0.001). In addition, the use of a higher dose of the contrast medium was associated with an increased hydration rate (*P* < 0.001). It is important to note that, compared with patients without concomitant diseases, the absolute saline volumes were significantly lower in patients with prior MI (915.25 ± 499.44 vs. 1122.16 ± 639.48 mL, *P* < 0.001), pre-existing renal disease (775.32 ± 680.36 vs. 1113.16 ± 628.44 mL, *P* < 0.001) and prior catheterization (901.60 ± 553.04 vs. 1130.25 ± 636.28 mL, *P* < 0.001), whereas it was higher in patients >75 years of age (1045.41 ± 566.61 vs. 1110.27 ± 635.64 mL, *P* = 0.014), in patients with hypertension (1143.47 ± 631.88 vs. 1051.44 ± 627.67 mL, *P* < 0.001), multi-vessel disease (1152.27 ± 619.52 vs. 1058.90 ± 640.24 mL, *P* < 0.001), left main lesion (1213.93 ± 704.35 vs. 1092.85 ± 621.76 mL, *P* < 0.001), left anterior descending branch lesion (1133.76 ± 626.63 vs. 1042.27 ± 638.45 mL, *P* < 0.001) and left circumflex branch lesion (1150.99 ± 626.74 vs. 1071.51 ± 633.34 mL, *P* < 0.001). There was no significant difference in the saline volumes between patients with and without reduced LVEF of <45%, single-vessel disease and right coronary lesion (Table 2).

### Trend of increased hydration use

**Figure 2:**
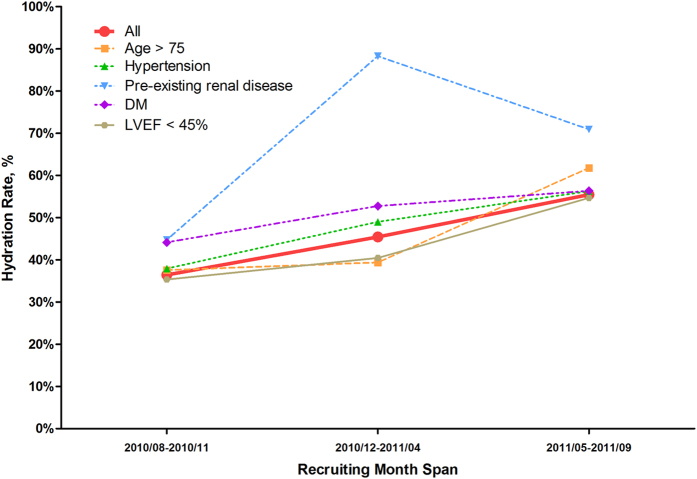
The trend of hydration rate over time in patients with different risk factors in three groups. Abbreviations: DM, diabetes mellitus; LVEF, left ventricular ejection fraction.

Figure 2 shows the trend of hydration use over time from August 2010 to September 2011. The overall rate of hydration use among patients undergoing CAG/PCI increased during the study period from 36.5% to 55.5%. Moreover, the rate of hydration therapy also increased over time in patients with one of the risk factors, such as pre-existing renal disease (44.7% to 70.9%), diabetes mellitus (44.1% to 56.3%), hypertension (37.9% to 56.3%), reduced LVEF (<45%; 35.3% to 54.7%) and older age (37.6% to 61.8%).

### Predictors of hydration

After adjusting for covariates described in baseline characteristics, hydration use was independently associated with older age, hypertension, diabetes mellitus, pre-existing renal disease, prior MI, STEMI, contrast dose, multi-vessel disease and LVEF <45% (Table 3). Patients aged >75 years (OR, 0.994; 95% CI, 0.991–0.997; *P* = 0.001) and with reduced LVEF (OR, 0.720; 95% CI, 0.614–0.844; *P* < 0.001) were less likely to receive hydration therapy, whereas patients with pre-existing renal disease (OR, 3.673; 95% CI, 2.694–5.008; *P* < 0.001) were at a higher risk of CIN and, hence, were more likely to receive hydration therapy. Similarly, patients with hypertension, diabetes mellitus, MI, STEMI, high contrast dose and multi-vessel disease were likely to receive hydration therapy due to a high risk of CIN.

## Discussion

To the best of our knowledge, this is the first study to provide contemporary information on the status of hydration use while undergoing CAG/PCI and its positive increase over time in Chinese patients, which is different from other retrospective study that analyzed the hydration practices in any patient undergoing coronary angiography or receiving specified contrast agent determined by cardiologists. The overall rate of hydration use remained relatively low (46.1%) in China. However, a significant increase in hydration use was observed over the study period in elderly patients and in patients with pre-existing renal diseases. In addition, multivariate analysis showed that hypertension, diabetes mellitus, pre-existing renal disease, prior MI, STEMI, contrast dose, multi-vessel disease and LVEF <45% were independent predictors of hydration use.

Different mechanisms are involved in the complex pathophysiology that underlies CIN. The direct toxic effects of the contrast medium would induce medullary vasoconstriction mediated by endothelin that reduce renal blood flow and damage the tubular epithelial cells and vascular endothelium, which lead to altered renal hemodynamics, regional hypoxia and the production of reactive oxygen radicals^9^,^10^,^19^. These may further increase tubular cell injury. In addition, the beneficial effects of saline hydration in salvaging CIN due to catheterization techniques are well documented^20^,^21^,^22^. The protective mechanisms underlying the salvaging effect have been investigated widely. Aurelio *et al*., reported that pre-procedural intravascular volume expansion could maintain medullary blood flow, enhance washout of the contrast medium through the kidney and shorten the time of contrast exposure, which might dilute the concentration of contrast medium, hampering the release of reactive oxygen species and cell necrosis factors that may damage the renal architecture. Moreover, hydration relieves regional hypoxia by preventing endothelial dysfunction and blood hyper viscosity by facilitating nitric oxide release^9^.

Despite the advancement in knowledge regarding CIN and hydration therapy, the awareness of the same in China remains weak. As mentioned previously, a low rate of hydration in patients of our study clearly suggests a weak understanding of both the disease and the therapy. A recent survey conducted by Prasad *et al*., to evaluate contemporary practice patterns with regard to prevention of CIN in patients undergoing invasive angiography from the Society of Cardiovascular Angiography and Intervention (SCAI) member cardiologists, revealed that 96.8% of the patients believe that the acute kidney injury (AKI) risk was always important; however, there were only 64.8% cardiologists who inclined to adopt the use of standardized volume expansion protocols to prevent the occurrence of CIN^23^.

The risk of CIN increases in patients with concomitant diseases. The patients with STEMI certainly have a higher risk of CIN^24^. Although current guidelines for the management of STEMI do not have a specific hydration strategy recommendation, a series of clinical trials have made some exploration. Jurado-Román *et al*., evaluated the effective role of peri-procedural intravenous hydration with saline in patients with STEMI undergoing primary PCI and observed an overall CIN incidence of 14% and reduction in the risk of CIN by 48% with the use of intravenous saline hydration peri-procedurally^21^. Furthermore, Maioli *et al*., suggested that the incidence of CIN would be lower with pre-procedural hydration strategy^25^. Previous studies have discovered that CIN occurred more frequently in patients treated with primary PCI than in those treated with elective procedures, with an incidence of up to 30%^26^. On the other hand, the rate and volume of hydration might be affected when patients presented active heart failure with pulmonary edema during acute coronary syndrome or STEMI. While in the present study, it was difficult to clarify this specific group of patients because we did not divide these patients before. Therefore, patients with STEMI should be emphasized on receiving hydration treatment. Our ongoing study (ATTEMPT, NCT02067195) aims to investigate the benefit and risk of aggressive hydration (faster and longer hydration) for patients with STEMI undergoing primary PCI^27^. The present study showed that 54.1% of the patients with STEMI received hydration, with a higher volume of hydration compared with patients without STEMI (mean, 1170 mL vs. 1096 mL). However, hydration was still deprived in 45.9% of the patients with STEMI, which warrants the need for awareness among clinicians and patients.

Furthermore, pre-existing renal disease based on personal health history is a major predictor of CIN in patients undergoing PCI/CAG procedures. The 2014 European Society of Cardiology Guidelines recommended patients with CKD to adopt hydration treatment 12 h before and at least 24 h after PCI^13^. Moreover, several clinical trials have proven that a higher volume of hydration efficiently reduces the risk of CIN and other adverse events^28^,^29^,^30^,^31^. In contrast, Liu *et al*., reported that excess hydration could not have a better effect on preventing CIN and may worsen heart failure^17^. The present study showed a higher hydration rate (77.4% vs. 45.7%) in high-risk patients with pre-existing renal disease than in patients with normal renal function, while the hydration volume was significantly lower. Our study also found that the volume of hydration was similar in patients with or without LVEF <45%, but a relatively lower hydration rate in patients with LVEF <45% (42.1% vs. 46.4%). In addition, hydration rate was increased with every 100-mL increase in the contrast dose used. These findings reflect the preliminary status of hydration therapy in Chinese patients undergoing CAG/PCI with different risk factors. However, the practice of hydration treatment is still not standardized and different from guideline recommendations at present in China.

Overall, the current study showed that the low rate of hydration treatment in patients undergoing CAG/PCI had gradually improved in China, especially in patients with risk factors such as pre-existing renal disease (44.7% to 70.9%, *P* < 0.001), diabetes mellitus (44.1% to 56.3%, *P* < 0.001), hypertension (37.9% to 56.3%, *P* < 0.001), reduced LVEF (35.3% to 54.7%, *P* < 0.001) and older age (37.6% to 61.8%, *P* < 0.001). This may be attributed to the promotion of coronary intervention technology and awareness of related complications, especially about CIN. Moreover, specialized CIN risk evaluation system, CIN education programs and hydration intensity monitoring have been established to improve awareness in patients undergoing CAG/PCI and clinicians on the benefits of hydration therapy.

Our study has several limitations: first, this was a post hoc observational analysis of a prospective observational study that focused on acute adverse drug reactions following iopromide administration without administration of prespecified hydration strategy; the study design may cause a bias due to the non-interventional nature and the variety of the quality of documentation of the patient’s hydration status across centers. Meanwhile, the prespecified contrast “iopromide” may increase the bias of strategy of prevention of CI-AKI and acute adverse drug reactions for operators. Second, several important indicators useful to evaluate the preventive effect of hydration on CIN, cannot be taken into account in a study design like that as additional lab tests are not conducted. Finally, most participating centers of this study were tertiary hospitals and therefore the data may not fully be representative of the whole country as clinical practice might differ to a large extend across hospitals in China.

In conclusion, the use of hydration among Chinese patients undergoing CAG/PCI which used to be relatively low in tertiary hospitals, including high-risk patients (e.g., pre-existing renal disease and older age) showed a significant increase in the study period. However, more awareness on hydration treatment and the risk of CIN in patients and clinicians along with an increase in hydration treatment compliance with guidelines among the high-risk patients are warranted in China.

## Methods

### Study design and participants

The present survey was a part of the TRUST study, which was a prospective, multicenter, observational study conducted on patients undergoing CAG or PCI procedure with the low-osmolar non-ionic contrast agent iopromide (Ultravist; Bayer, Berlin, Germany) at 63 hospitals in China from August 2010 to September 2011 (ClinicalTrials.gov identifier: NCT01206257). The objectives of the present analysis were to provide information on intravenous hydration practice in China, identify any changes over time and factors associated with hydration use, as well as to understand opportunities for further improvement. The study protocol conformed to the principles of the Declaration of Helsinki and was approved by the Chinese ethics committee of registering clinical trials (ChiECRCT), China (approval number ChiECRCT-2010018, 31^st^ August 2010) as the leading ethics board and additionally was approved by the local ethics boards of each participating center.

The study was approved by local ethics committees and We conducted a post hoc analysis of patient data from the TRUST study^18^. Patients with coronary artery disease scheduled for CAG and/or PCI with iopromide (300 or 370 mg iodine/mL) were included in the study, and a written informed consent was procured from all the eligible patients. Pregnant and lactating women, patients with a contraindication to iopromide and/or cardiac catheterization were excluded from the study.

### Study procedures

#### Hydration treatment

We recorded whether patients accepted intravenous isotonic saline hydration pre or post-procedurally or not and the total hydration volume through the case report form. All the enrolled patients were stratified into two groups based on their hydration status (those who received the hydration treatment and those who did not receive the hydration treatment).

#### Coronary angiography or PCI

Coronary angiography was performed using standard guide catheters, guide wires, balloon catheters and stents by the femoral or radial approach according to the standard clinical practice. The concentration and dose of intra-arterially injected iopromide were left to the discretion of the interventional cardiologist according to operative requirements.

#### Screening protocol and assessment criteria

Participants were asked to complete a questionnaire documenting their socio-demographic status (e.g., age and sex), personal and family health history (e.g., hypertension, diabetes and pre-existing renal disease), physical examination (e.g., weight and blood pressure) and laboratory examination (e.g., serum lipids) with the assistance of trained nurses. Medical records of the patients were also reviewed by the nurses to confirm hydration treatment during the perioperative period. Data testing and collection were conducted at the local central hospital.

#### Statistical analysis

All the analyses were conducted using the SPSS 22.0 software (IBM Corp, Armonk, New York, United States). Continuous variables are presented as mean ± standard deviation, and categorical variables are expressed as counts and percentages with 95% confidence intervals. Differences in demographic and socio-economic characteristics, presence of metabolic conditions, clinical presentation and features of coronary artery among participants were analyzed using two-tailed unpaired student *t*-tests for continuous variables and chi-square tests for categorical variables. A multivariable logistic regression model adjusted for age (>75 years), hypertension, diabetes mellitus, pre-existing renal disease, prior myocardial infarction (MI), ST-elevation MI (STEMI), contrast dose, multi-vessel disease and left ventricular ejection fraction (LVEF <45%) was used to identify factors independently associated with hydration use. A *P* value of <0.05 was considered to be statistically significant.

## Additional Information

**How to cite this article:** Bei, W. *et al*. Post-Hoc Study: Intravenous Hydration Treatment in Chinese Patients with High Risk of Contrast-Induced Nephropathy Following Percutaneous Coronary Intervention. *Sci. Rep.* **7**, 45023; doi: 10.1038/srep45023 (2017).

**Publisher's note:** Springer Nature remains neutral with regard to jurisdictional claims in published maps and institutional affiliations.

## Figures and Tables

**Table 1:** Baseline and procedural characteristics of patients in the hydration and non-hydration groups.

Characteristics	Total (N = 17139)	Hydration (N = 7901)	Non-hydration (N = 9238)	*P* value
Age (years)	60.33 ± 10.38	60.24 ± 10.31	60.41 ± 10.44	0.285
Age >75 years, n (%)	1139 (6.8)	505 (6.5)	634 (7.0)	0.228
Female, n (%)	6117 (35.7)	2829 (35.8)	3288 (35.6)	0.783
Weight (kg)	69.16 ± 10.71	70.05 ± 11.02	68.41 ± 10.38	<0.0001
Previous history				
History of hypertension, n (%)	9528 (55.6)	4619 (58.5)	4909 (53.1)	<0.0001
History of diabetes mellitus, n (%)	3441 (20.1)	1778 (22.5)	1663 (18.0)	<0.0001
Prior MI, n (%)	1187 (6.9)	646 (8.2)	541 (5.9)	<0.0001
Pre-existing renal disease, n (%)	239 (1.4)	185 (2.3)	54 (0.6)	<0.0001
Family history of CAD, n (%)	1185 (6.9)	660 (8.4)	525 (5.7)	<0.0001
Prior cardiac catheterization, n (%)	1749 (10.2)	864 (10.9)	885 (9.6)	0.003
Systolic blood pressure (mmHg)	134.20 ± 18.46	135.04 ± 18.66	133.47 ± 18.25	<0.0001
Diastolic blood pressure (mmHg)	80.21 ± 11.48	81.31 ± 11.75	79.27 ± 11.16	<0.0001
Preoperative metformin, n (%)	319 (1.9)	168 (2.1)	151 (1.6)	0.018
LVEF < 35%, n (%)	158 (0.9)	70 (0.9)	88 (1.0)	0.628
LVEF <45%, n (%)	717 (4.2)	302 (3.8)	315 (4.6)	0.025
Clinical presentation				
STEMI, n (%)	1890 (11.0)	1023 (12.9)	867 (9.4)	<0.0001
NSTEMI, n (%)	833 (4.9)	375 (4.7)	458 (5.0)	0.521
Unstable angina, n (%)	8689 (50.7)	4610 (58.3)	4079 (44.2)	<0.0001
Stable angina, n (%)	2039 (11.9)	628 (7.9)	1411 (15.3)	<0.0001
Others, n (%)	3912 (22.8)	1388 (17.6)	2524 (27.3)	<0.0001
TC (mmol/L)	4.51 ± 1.17	4.57 ± 1.17	4.46 ± 1.17	<0.0001
TG (mmol/L)	1.69 ± 0.95	1.72 ± 0.98	1.66 ± 0.91	<0.0001
Contrast dose (mL)	124.80 ± 72.88	140.38 ± 80.59	111.49 ± 62.57	<0.0001
Coronary artery features				
Normal, n (%)	3044 (17.8)	1036 (13.1)	2008 (21.7)	<0.0001
Single-vessel disease, n (%)	6756 (39.4)	2943 (37.2)	3813 (41.3)	<0.0001
Multi-vessel disease, n (%)	7339 (42.8)	3922 (49.6)	3417 (37.0)	<0.0001
Left main disease, n (%)	1456 (8.5)	809 (10.2)	647 (7.0)	<0.0001
Left anterior descending branch, n (%)	11,142 (65.0)	5434 (68.8)	5708 (61.8)	<0.0001
Left circumflex artery, n (%)	6478 (37.8)	3354 (42.5)	3124 (33.8)	<0.0001
Right coronary artery, n (%)	6534 (38.1)	3359 (42.5)	3175 (34.4)	<0.0001
Stents implanted, n (%)	8287 (48.4)	4078 (51.6)	4209 (45.6)	<0.0001
Number of stents implanted	1.68 ± 0.76	1.69 ± 0.76	1.67 ± 0.76	0.302

Abbreviations: MI, myocardial infarction; CAD, coronary artery disease; LVEF, left ventricular ejection fraction; STEMI, acute ST segment elevation myocardial infarction; NSTEMI, non-ST segment elevation acute myocardial infarction; TC, total cholesterol; TG, total triglyceride.

**Table 2:** Hydration status of high-risk patients.

Characteristics	Yes	No	*P* value
Age >75 years	1045.41 ± 566.61	1110.27 ± 635.64	0.014
Hypertension	1143.47 ± 631.88	1051.44 ± 627.67	<0.001
Diabetes mellitus	1112.49 ± 600.26	1103.15 ± 640.60	0.570
Prior MI	915.25 ± 499.44	1122.16 ± 639.48	<0.001
Pre-existing renal disease	775.32 ± 680.36	1113.16 ± 628.44	<0.001
Prior cardiac catheterization	901.60 ± 553.04	1130.25 ± 636.28	<0.001
LVEF <45%	1104.78 ± 632.81	1131.75 ± 601.46	0.446
STEMI	1170.44 ± 674.98	1095.55 ± 624.51	0.001
NSTEMI	1177.60 ± 604.46	1101.64 ± 632.88	0.023
Unstable angina	1087.17 ± 642.63	1130.59 ± 615.33	0.002
Stable angina	1061.78 ± 574.41	1109.00 ± 636.33	0.050
Contrast dose ≥100 mL	1078.68 ± 660.64	1166.69 ± 554.46	<0.001
Contrast dose ≥200 mL	1000.65 ± 610.97	1142.91 ± 634.88	<0.001
Contrast dose ≥300 mL	1121.22 ± 632.90	1103.90 ± 631.65	0.515
Single-vessel disease	1098.69 ± 656.55	1109.14 ± 616.56	0.484
Multi-vessel disease	1152.27 ± 619.52	1058.90 ± 640.24	<0.001
Left main lesion	1213.93 ± 704.35	1092.85 ± 621.76	<0.001
Left anterior descending branch lesion	1133.76 ± 626.63	1042.47 ± 638.45	<0.001
Left circumflex branch lesion	1150.99 ± 626.74	1071.51 ± 633.34	<0.001
Right coronary lesion	1106.29 ± 593.58	1104.48 ± 658.58	0.898

Abbreviations: MI, myocardial infarction; LVEF, left ventricular ejection fraction; STEMI, ST segment elevation myocardial infarction; NSTEMI, non-ST segment elevation acute myocardial infarction.

**Table 3:** Multivariable analysis of factors associated with hydration therapy in patients undergoing CAG/PCI.

Risk factors	Multivariate logistic regression		
	OR	95% CI	*P* value
Age >75 years	0.994	0.991–0.997	<0.001
Hypertension	1.192	1.118–1.270	<0.001
Diabetes mellitus	1.182	1.093–1.278	<0.001
Pre-existing renal disease	3.673	2.694–5.008	<0.001
Prior MI	1.187	1.048–1.343	0.007
STEMI	1.168	1.056–1.292	0.003
Contrast dose	1.005	1.004–1.005	<0.001
Multi-vessel disease	1.293	1.208–1.384	<0.001
LVEF <45%	0.720	0.614–0.844	<0.001

Abbreviations: MI, myocardial infarction; LVEF, left ventricular ejection fraction; STEMI, ST segment elevation myocardial infarction.
